# Effects of feeding a *Bacillus*-based direct-fed microbial during a 60-day receiving program on performance and physiological responses of feeder cattle

**DOI:** 10.1093/tas/txag063

**Published:** 2026-05-12

**Authors:** Arnaldo C Limede, Rodrigo S Marques, Fernando A A Cidrini, Erollykens F Santos, Gilyard A P Souza, Izadora S Souza, Reinaldo F Cooke, Bruno I Cappellozza

**Affiliations:** School of Animal Sciences, Virginia Polytechnic Instituteand State University, Blacksburg, VA, 24061, United States; School of Animal Sciences, Virginia Polytechnic Instituteand State University, Blacksburg, VA, 24061, United States; School of Animal Sciences, Virginia Polytechnic Instituteand State University, Blacksburg, VA, 24061, United States; School of Animal Sciences, Virginia Polytechnic Instituteand State University, Blacksburg, VA, 24061, United States; Departamento de Zootecnia, Universidade Estadual Paulista “Júlio de Mesquita Filho” (UNESP), Jaboticabal, São Paulo, 14884-900, Brazil; Department of Animal Science, Texas A&M University, College Station, TX 77845, United States; Department of Animal Science, Texas A&M University, College Station, TX 77845, United States; Novonesis, Lyngby, 2800, Denmark

**Keywords:** *Bacillus* spp, direct-fed microbials (DFM), feedlot receiving, performance, stress mitigating

## Abstract

The objective was to evaluate the effects of *Bacillus*-based DFM supplementation during a 60-day feedlot receiving phase. Eighty-four Angus calves were ranked by initial body weight (**BW = **280 ± 7.4 kg), age (233 ± 6 days), and sex (38 heifers and 46 steers), and allocated to 20 pens in a completely randomized design. Pens were randomly assigned to receive (i) a total mixed ration (**TMR**) plus 165 g of protein-mineral supplement + 3 g of a *Bacillus*-based DFM containing *Bacillus licheniformis* 809 and *B. subtilis* 810 (**BAC**; *n* = 10; 2.2 × 10^9^ CFU of the mixture/g; Bovacillus, Novonesis, Lyngby, Denmark), and (ii) a TMR plus 165 g of protein-mineral supplement without DFM (**CON**; *n* = 10). Drylot pens were considered the experimental unit, with 4 to 5 calves per pen. Animals had free-choice access to the TMR offered daily at 0800 h. Feed samples were collected daily, and the refusals were removed weekly from the feed bunks. All samples were weighed, sampled, and dried to calculate dry matter intake (**DMI**). Calves shrunk BW was recorded on days 0 and 60 after 16 h fasting from water and feed. These values were used to calculate the average daily gain (**ADG**). Feed efficiency was calculated as the total experimental period BW gain divided by the total DMI for each pen. Blood samples were collected on days 0, 7, 14, 21, 28, 42, and 56 and analyzed for plasma concentrations of cortisol, nonesterified fatty acids (**NEFA**), β-hydroxybutyrate (**BHBA**), and glucose. Plasma samples from days 0, 7, 14, and 21 were also analyzed for haptoglobin concentrations. Hair samples were collected from the tail switch on days 0, 14, 28, and 42 and analyzed for cortisol concentrations. All data were analyzed using the MIXED procedure of SAS. Treatment was included as a fixed effect and pen(treatment) as a random effect; repeated-measures included time, treatment × time interaction, and calf(pen) as a random effect. There was a tendency for increased DMI in the BAC group compared to the CON group (*P* = 0.06), without any effect on the remaining performance parameters (*P* ≥ 0.50). A treatment × day interaction was observed for plasma cortisol concentration (*P* < 0.01), where animals from the CON group had increased cortisol levels on day 14. Mean plasma haptoglobin concentration was greater in CON compared to BAC cohorts (*P* = 0.03). Collectively, these findings suggest that *Bacillus*-based DFM enhanced feed intake and reduced plasma stress markers in feeder calves during a 60-day receiving program.

## Teaser

Supplementing feeder calves with a *Bacillus*-based direct-fed microbial tended to increase feed intake and reduce biological markers of stress and inflammation during the feedlot receiving period.

## Introduction

Feeder cattle are subject to a sequence of stressors, such as weaning, transport, commingling, and diet changes upon feedlot arrival (Loerch and Fluharty 1999; Duff and Galyean 2007; Wernicki et al. 2014; Galyean et al. 2022). The implication of these practices may impose significant stress on the animals, resulting in changes in innate immunity, including inflammation and acute reactions (Carroll and Forsberg 2007; Cooke 2017). Consequently, extensive research has investigated management and nutritional interventions aimed at mitigating morbidity and optimizing physiological and productive responses in feeder calves during the receiving period (Duff and Galyean 2007; Galyean et al. 2022).

The animal industry has experienced increasing pressure to enhance production efficiency and animal health, minimize environmental impacts, and ensure product safety (Ban and Guan 2021). The use of biotics, also known as probiotics or direct-fed microbials (**DFM**), may improve nutrient digestion, decrease morbidity and mortality rates, mitigate stress-induced behaviors, and enhance growth rate and efficiency (Swanson et al. 2025). *Bacillus* spp. is a promising DFM for animal production because it survives and remains viable after feed preparation (Cappellozza et al. 2025), and increase nutrient degradability in vitro (Pan et al. 2022; Cappellozza et al. 2023), total-tract nutrient digestibility (Limede A. C. et al. 2025; De Souza et al. 2026), feed efficiency in the feedlot finishing phase (Dias et al. 2022; Cordeiro et al. 2024), and decreased morbidity in high-risk feeder cattle (Mackey et al. 2024).

The feedlot receiving period is recognized as one of the most physiologically and nutritionally challenging stages of the beef production cycle (Duff and Galyean 2007; Galyean et al. 2022); therefore, evaluation of *Bacillus-*based DFM supplementation during this period is warranted. Hence, we hypothesized that this nutritional strategy would enhance feeder calves’ immunity and intake and, subsequently, overall performance during a 60-day feedlot receiving program. To test this hypothesis, this experiment evaluated the performance, health, and physiological responses of feeder calves supplemented with a *Bacillus*-based DFM during a 60-day feedlot receiving period.

## Material and methods

This study was conducted at the Virginia Polytechnic Institute and State University—Kentland Farms. Experimental procedures involving animals were reviewed and approved by the Virginia Tech Institutional Animal Care and Use Committee (protocol #24–091).

### Animals, experimental design, and diets

Eighty-four Angus calves were obtained from Virginia Polytechnic and State University, Kentland Farms (Blacksburg, VA; 37°11′59″N 80°33′53″W, 508 m), and transported to the feedlot facility located at the same farm (day -1). Upon arrival, calves were treated with an antiparasitic and vaccinated with a clostridial and a respiratory vaccine. Animals received a topical application of *Cydectin™* (moxidectin; Boehringer Ingelheim Animal Health USA Inc., Duluth, GA, USA) at 1 mL/10 kg of BW along the dorsal midline, followed by a 2-mL subcutaneous injection of *BOVILIS Vision® 7 with SPUR* (Merck Animal Health, Madison, NJ, USA), containing *Clostridium chauvoei*, *C. septicum*, *C. novyi*, *C. sordellii*, and *C. perfringens* types C and D bacterin–toxoid. Cattle also received 2 mL SC of *Pyramid® 5 + Presponse® SQ* (Boehringer Ingelheim Animal Health USA Inc., Duluth, GA, USA), containing modified-live infectious bovine rhinotracheitis virus, bovine viral diarrhea virus types 1 and 2, parainfluenza-3 virus, bovine respiratory syncytial virus, and *Mannheimia haemolytica* toxoid.

On day 0, calves were allocated to a collective drylot pen without access to water and feed for 16-h before body weight (**BW**) was recorded. Following, calves were ranked by initial **BW** (280 ± 7.4 kg), age (233 ± 6 days), and sex (38 heifers and 46 steers), and allocated to 20 pens (10 pens per treatment) in a completely randomized design. Pens (5 × 9 m; 2.5 m of linear feed bunk) were assigned to receive (i) a total mixed ration (**TMR**) plus 165 g of protein-mineral supplement + 3 g of a *Bacillus*-based DFM containing *Bacillus licheniformis* 809 and *B. subtilis* 810 (**BAC**; *n* = 10; 2.2 × 10^9^ CFU of the mixture/g; Bovacillus, Novonesis, Lyngby, Denmark), or (ii) a TMR plus 165 g of protein-mineral supplement without DFM (**CON**; *n* = 10). Animals were kept in drylot pens containing 4 to 5 calves per pen (sixteen pens contained 4 animals [2 heifers and 2 steers], and 4 pens contained 5 [either 2 heifers and 3 steers or 3 heifers and 2 steers]), with similar initial BW, age, and sex between treatments.

The treatment supplement consisted of 115 g of a commercial mineral mix (Table 1) combined with 50 g of dried distillers’ grains per calf and was administered as a top-dressing immediately after the TMR delivery. Animals had free-choice access to a TMR with a 50:50 roughage-to-concentrate ratio, balanced using the NASEM (2016) software, and offered daily at 0800 h. Ingredients, composition, and nutritional profile are shown in Table 1.

**Table 1:** Composition and nutritional profile of experimental TMR and supplements.

Item	TMR^a^	Supplement^b^
**Composition, % DM basis**		
**Corn silage**	53.5	…
**Ground corn**	22.6	…
**Dry corn gluten feed**	23.9	…
**Dry distillers’ grain**	…	30.3
**Mineral mix**^c^	…	69.7
**Nutritional Profile, %DM basis**		
**Crude protein**	12.7	8.8
**Neutral detergent fiber**	30.5	9.0
**Acid detergent fiber**	15.9	10.4
**Ether extract**	3.2	4.8
**Ash**	5.42	71.4
**Total digestible nutrients**	73.0	26.0

a Animals had free-choice access to a TMR balanced using the NASEM (2016) software, and offered daily at 0800 h.

b The supplement was used as the treatment carrier, which consisted of 115 g of a commercial mineral mix combined with 50 g of dried distillers’ grains. The supplement was offered as a top dressing and was fully consumed within 30 minutes.

c Containing 9.0% Ca, 4.0 P, 16.4% NaCl, 14% Mg, 1500 ppm Cu, 1300 ppm Zn, 20 ppm Se, 330,000 IU/kg of vitamin A, 33,000 IU/kg of vitamin D3, and 880 IU/kg of vitamin E.

### Sampling and laboratory analyses

Samples of TMR and its ingredients were collected weekly, pooled across weeks, and sent to a commercial laboratory (Dairy One Forage Laboratory, Ithaca, NY) for chemical composition analysis. Feed samples were collected daily and dried in an oven for 72 h at 55 °C. Once a week, the refusals were removed from the feed bunks, weighed, sampled, and dried using the same procedure used for feed samples. Feed intake for each pen was calculated by subtracting weekly refusals from the weekly total offered, dividing by the number of calves per pen, and expressing the result as kg per calf/day. Individual calf BW was recorded on days 0 (initial BW) and 60 (final BW) after 16 h of fasting from water and feed and used to calculate average daily gain (**ADG**). The feed efficiency was calculated by dividing the total experimental period BW gain by the total experimental dry matter intake (**DMI**) from each pen.

Blood samples were collected on days 0, 7, 14, 21, 28, 42, and 56 via jugular venipuncture into commercial collection tubes (Vacutainer, 10 mL; Becton Dickinson, Franklin Lakes, NJ) containing or not freeze-dried sodium heparin for plasma or serum collection, respectively. All blood samples were collected prior to daily feeding, placed immediately on ice, centrifuged (2500 × *g* for 30 min; 4 °C), and stored at −80 °C. All blood samples were analyzed for concentrations of cortisol (radioimmunoassay kit #07,221,106, MP Biomedicals, Santa Ana, CA; Colombo et al. 2019), nonesterified fatty acids (**NEFA**), β-hydroxybutyrate (**BHBA**), and glucose (Carysta High Volume Chemistry Analyzer; Zoetis; Colombo et al. 2021). Plasma samples from days 0, 7, 14, and 21 were analyzed for haptoglobin concentrations (Cooke and Arthington 2013). The intra- and inter-assay CVs across all analyses were ≤ 4.1%. Hair samples were collected from the tail switch of steers on days 0, 14, 28, and 42 and analyzed for cortisol concentrations using ELISA kit (High Sensitivity 1-E3002; Salimetrics Expanded Range, State College, PA, USA) as described in Schubach et al. (2017) with an intra-assay and inter-assay CV of 3.24% and 2.84%, respectively.

### Statistical analysis

All performance and physiological results were analyzed using the pen as the experimental unit (*n* = 10 pens per treatment), the MIXED procedure of SAS (SAS Inst. Inc., Cary, NC), and the Satterthwaite approximation to determine the denominator degrees of freedom for tests of fixed effects. These data were analyzed using pen(treatment) and calf(pen) as random variables, but for intake and feed efficiency were used pen(treatment) as the random variable as described by Colombo et al. (2020), Schubach et al. (2020), and Kvamme et al. (2024). Model statements for DMI and blood parameters contained the fixed effects of treatment, week/day, and their interactions. Model statements for BW parameters and feed efficiency contained the effects of treatment. The specified term for all repeated statements was pen(treatment) for DMI and calf(treatment) for all other analyses. The covariance structure used was first-order autoregressive, which provided the smallest Akaike information criterion and, hence, the best fit for all variables analyzed. Results were reported as least square means or covariate-adjusted least square means for blood and exit velocity variables. Significance was set at *P* ≤ 0.05, and tendencies were determined if *P* > 0.05 and ≤ 0.10.

## Results and discussion

### Performance

No treatment × week interaction was detected for DMI (*P* = 0.69; Table 2). However, there was a tendency for greater DMI in the BAC group compared with the CON group (*P* = 0.06). In agreement with our results, supplementation with the same *Bacillus*-based DFM increased DMI in Angus cows consuming forage-based diets (Limede A. C. et al. 2025) as well as silage-based diets (Limede A. et al. 2025). The increase in DMI may be partially explained by in vitro findings from Cappellozza et al. (2023) and Pan et al. (2022), in which incubations of *Bacillus licheniformis* 809 and *B. subtilis* 810 with commercial dairy TMRs increased dry matter and neutral detergent fiber degradability. *Bacillus* spp. is known to produce a wide variety and quantity of enzymes, establishing them as important enzyme-producing microorganisms (Schallmey et al. 2004; Luise et al. 2022). Their ability to improve dietary carbohydrate and protein utilization (Sun et al. 2013; Souza et al. 2017), ruminal DM and NDF disappearance, and total-tract apparent nutrient digestibility (Limede A. C. et al. 2025; De Souza et al. 2026) might also contribute to enhanced DMI.

**Table 2:** Performance parameters of calves during a 60-day feedlot receiving phase fed (i) a total mixed ration (TMR) plus 165 g of protein-mineral supplement + 3 g of a *bacillus*-based DFM containing *Bacillus licheniformis* and *B. subtilis* (**BAC**; *n* = 10; 2.2 × 10^9^ CFU of the mixture/g; bovacillus, novonesis, lyngby, Denmark), and (ii) a TMR plus 165 g of protein-mineral supplement without DFM (**CON**; *n* = 10).

Item	Treatments		SEM	*P-*value		
	CON	BAC		Treatment	Week	T × W
**Dry Matter Intake, kg/day**	6.67	7.01	0.12	0.06	<0.01	0.69
**Initial Body Weight, kg** ^a^	280.9	280.2	5.01	0.91	…	…
**Final Body Weight, kg** ^a^	332.0	335.9	4.42	0.54	…	…
**Average Daily Gain, kg/day** ^b^	0.917	0.996	0.89	0.53	…	…
**Feed Efficiency, g/kg of DMI** ^c^	137.5	147.2	14.2	0.50	…	…

a Recorded after 16 h fasting of water and feed.

b Calculated as the difference between final and initial body weight divided by the number of experimental days.

c Calculated by dividing the total experimental period BW gain by the total experimental DMI from each pen.

Despite changes in the DMI observed in the present study, there were no treatment effects on final BW (*P* = 0.54), ADG (*P* = 0.53), or feed efficiency (*P* = 0.50). Contrary to our findings, improvements in feed efficiency with *Bacillus-*based DFM supplementation have been reported during the feedlot finishing phase. Dias et al. (2022) reported that cattle fed for 115 days had 5% greater feed efficiency when receiving *Bacillus*-based DFM compared with the control (no DFM). Similarly, Cordeiro et al. (2024) observed a 6.4% increase in feed efficiency when supplementing animals with the same DFM for 84 days. It is important to note that the present study was limited to a 60-day evaluation period, which may not have been sufficient to detect measurable improvements in growth performance resulting from increased DMI and may help explain discrepancies with previous results.

### Blood parameters and hair cortisol concentration

**Figure 1:**
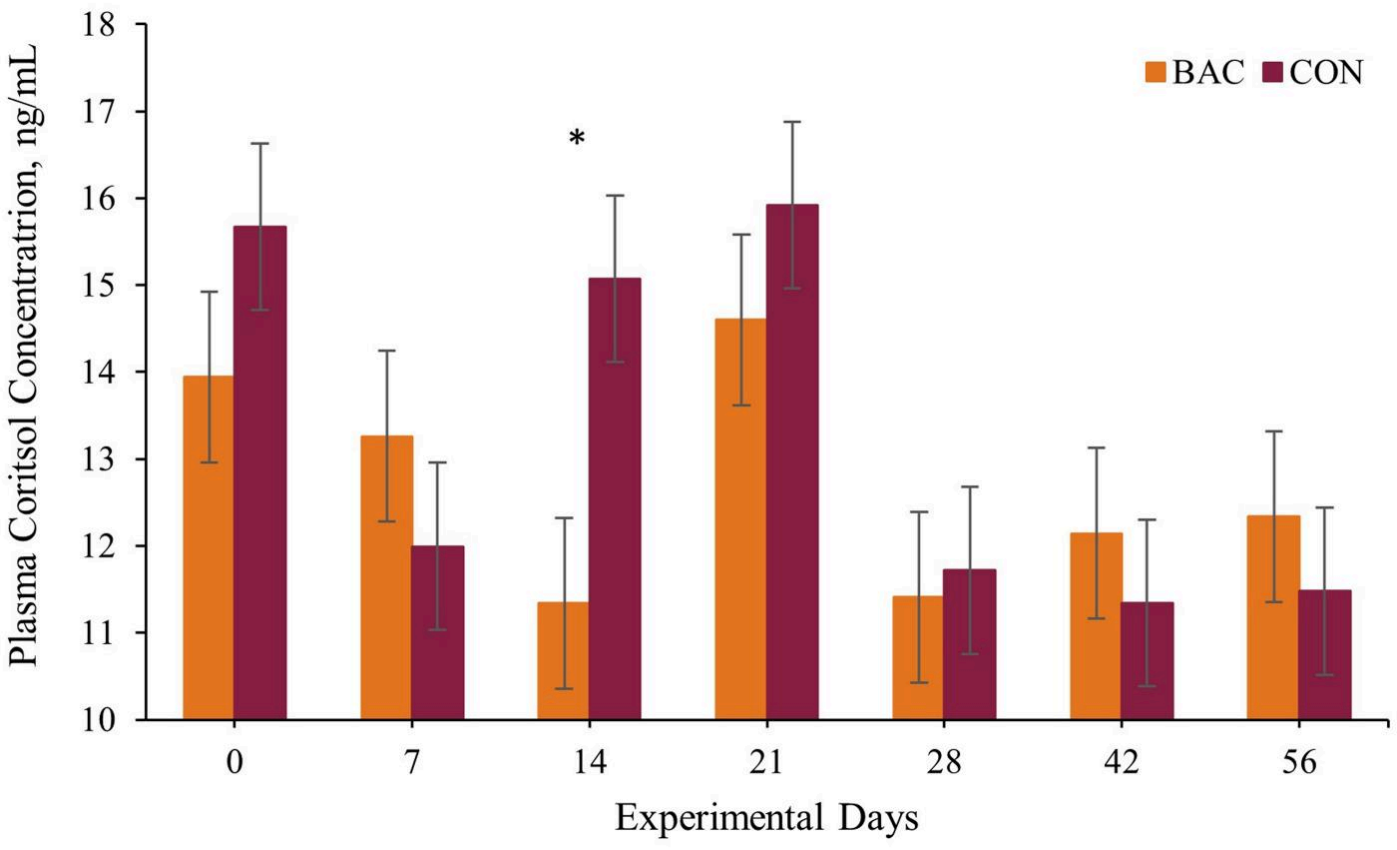
Plasma cortisol concentration during a 60-day feedlot receiving phase from calves receiving (i) a total mixed ration (TMR) plus 165 g of protein-mineral supplement + 3 g of a *bacillus*-based DFM containing *Bacillus licheniformis* and *B. subtilis* (BAC; *n* = 10; 2.2 × 10^9^ CFU of the mixture/g; bovacillus, novonesis, lyngby, Denmark), and (ii) a TMR plus 165 g of protein-mineral supplement without DFM (CON; *n* = 10). within treatment, *P ≤ 0.05.

There was a treatment × day interaction for plasma cortisol concentration (*P* = 0.01; Table 3). Animals from the CON group showed elevated plasma cortisol concentration compared to the BAC group on day 14 (Fig. 1). Plasma cortisol levels are considered indicators of stress (Carroll and Forsberg 2007), increasing when the hypothalamic–pituitary–adrenal axis becomes active, regardless of whether the stressor is psychological, physiological, or physical (Cooke 2017). Thus, the elevated plasma cortisol observed in CON on day 14 of the experimental period may be associated with increased stress at that time point. This finding is supported by the increased mean plasma haptoglobin concentration (*P* = 0.03), which was 17% higher in the CON group than that observed in the BAC group, indicating that supplementation with *Bacillus*-based DFM may mitigate the stress-associated increase in acute-phase protein response (Cooke 2017). Additionally, these results may be associated with the increased DMI observed in the BAC group, as the acute-phase protein response is negatively correlated with feed intake in beef cattle (Araujo et al. 2010). *Bacillus* spp. supports the lower gut health by stimulating mucin production (Santano et al. 2020), by competing with potentially harmful bacteria for binding sites through the formation of biofilm (Copani et al. 2020), and by maintaining the integrity of the intestinal wall by strengthening the gut barrier (Rhayat et al. 2019). Furthermore, supplementation with *Bacillus subtilis* enhances immunity by activating innate immune cells, increasing T- and B-cell responses, regulating cytokine production, and boosting antibody formation (Sun et al. 2013). The release of proinflammatory cytokines during the acute-phase protein response, such as IL-1, IL-6, and tumor necrosis factor-α (**TNF-α**), may affect the DMI by modulating the central nervous and endocrine systems, which in turn affect gastrointestinal tract motility and flow (Klasing and Korver 1997; Gouvêa et al. 2022), and help to explain the DMI and the stress-related responses observed herein. Accordingly, Mackey et al. (2025) reported that beef heifers challenged with LPS and supplemented with *B. licheniformis* 809 and *B. subtilis* 810 had an alleviated acute-phase response, faster DMI resumption post-LPS, and also a lower blood mRNA expression of genes associated with oxidative stress. Additionally, calves born from pregnant heifers and multiparous cows fed *B. licheniformis* 809 and *B. subtilis* 810 had greater antibody response following vaccination against BRD pathogens (Izquierdo et al. 2024; Moriel et al. 2025). Collectively, these outcomes represent key mechanisms for modulating the inflammatory response in livestock animals, directly impacting DMI and performance (Gouvêa et al. 2022).

**Table 3:** Blood parameters and hair cortisol concentration of calves during a 60-day feedlot receiving phase fed (i) a total mixed ration (TMR) plus 165 g of protein-mineral supplement + 3 g of a *bacillus*-based DFM containing *Bacillus licheniformis* and *B. subtilis* (**BAC**; *n* = 10; 2.2 × 10^9^ CFU of the mixture/g; bovacillus, novonesis, lyngby, Denmark), and (ii) a TMR plus 165 g of protein-mineral supplement without DFM (**CON**; *n* = 10).

Item	Treatments		SEM	*P*-value		
	CON	BAC		Treatment	Day	T × D
**Plasma glucose, mg/dL^a^**	78.0	80.3	1.58	0.32	<0.01	0.44
**NEFA, mEq/L** ^a^	0.06	0.07	0.003	0.25	<0.01	0.84
**BHBA, mg/dL** ^a^	2.80	2.77	0.12	0.87	<0.01	0.37
**Cortisol, ng/mL** ^a^	13.3	12.7	0.69	0.54	<0.01	0.01
**Haptoglobin, mg/dL** ^b^	0.33	0.28	0.01	0.03	<0.01	0.86
**Hair cortisol, pg/mg** ^c^	4.22	4.23	0.18	0.98	<0.01	0.73

a Plasma samples from experimental days 0, 7, 14, 21, 28, 42, and 56 were analyzed for cortisol (radioimmunoassay kit #07,221,106, MP Biomedicals, Santa Ana, CA; Colombo et al. 2019), NEFA, and BHBA (Carysta High Volume Chemistry Analyzer; Zoetis; Colombo et al. 2021).

b Plasma samples from days 0, 7, 14, and 21 were analyzed for haptoglobin concentrations (Cooke and Arthington 2013).

c Hair samples were collected from the tail switch of steers on days 0, 14, 28, and 42 and analyzed for cortisol concentrations as in Schubach et al. (2017).

**Figure 2:**
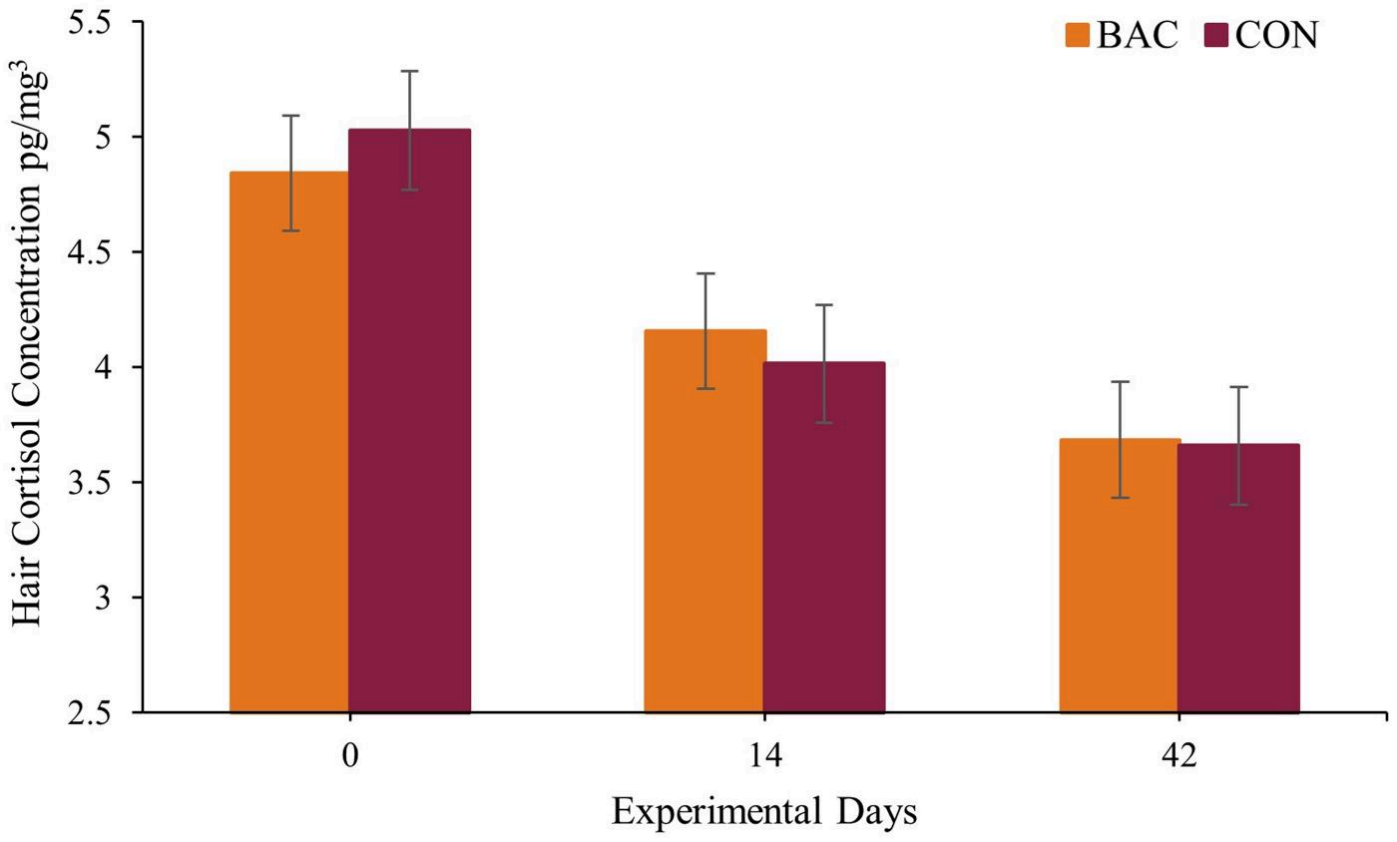
Hair cortisol concentration during a 60-day feedlot receiving phase from calves receiving (i) a total mixed ration (TMR) plus 165 g of protein-mineral supplement + 3 g of a *bacillus*-based DFM containing *Bacillus licheniformis* and *B. subtilis* (BAC; *n* = 10; 2.2 × 10^9^ CFU of the mixture/g; bovacillus, novonesis, lyngby, Denmark), and (ii) a TMR plus 165 g of protein-mineral supplement without DFM (CON; *n* = 10).

Although differences were observed in plasma cortisol and haptoglobin concentrations, no effects were detected on plasma glucose, NEFA, or BHBA (*P* ≥ 0.25), nor on hair cortisol concentration (*P* = 0.98). Elevated NEFA, BHBA, and glucose are typically associated with negative energy balance (Li et al. 2016), a condition not observed in the present study, as cattle in both treatment groups exhibited similar BW gain, indicative of positive energy balance. Despite the greater circulating stress markers in the CON group, which could negatively impact the animal performance (Gouvêa et al. 2022), these animals likely maintained sufficient feed intake to ensure adequate energy supply for the maintenance of homeostasis. Assessing cortisol concentration in hair is a simple, non-invasive technique for evaluating cortisol changes over long-term periods (Moya et al. 2013). Animals from both treatments showed elevated hair cortisol concentration at the beginning of the experimental period (Fig. 2, *P* < 0.01), which may be associated with cumulative stress events such as weaning and transport before the feedlot entry (Carroll and Forsberg 2007; Wernicki et al. 2014; Cooke 2017). Measuring hair cortisol concentration is considered an effective biomarker for chronic health conditions, as it avoids reflecting short-term cortisol fluctuations found in plasma and shows little correlation with blood markers like haptoglobin, BHBA, and glucose (Burnett et al. 2015), which might explain the lack of treatment effects observed in the present study.

## Conclusion

Supplementation with a *Bacillus*-based DFM tended to increase DMI, although overall performance was not affected during a 60-day experimental period. Notably, *Bacillus* supplementation decreased plasma cortisol and haptoglobin concentrations, suggesting a potential mitigation of stress and acute-phase protein responses. Collectively, these findings suggest that while *Bacillus*-based DFM may enhance feed intake and reduce markers of stress and inflammation, a longer supplementation period may be necessary to observe significant improvements in growth performance.

## List of abbreviations

ADG: average daily gain
BHBA: β-hydroxybutyrate
BW: body weight
DFM: direct-fed microbials
DM: dry matter
DMI: dry matter intake
NDF: neutral detergent fiber
NEFA: nonesterified fatty acids
TMR: total mixed ration
TNF-α: tumor necrosis factor-α
